# Medicaid Expansion and Patient Safety Culture in USA Hospitals

**DOI:** 10.3390/ijerph22121795

**Published:** 2025-11-27

**Authors:** Jayson Forbes, Alejandro Arrieta

**Affiliations:** 1 Public Health Department, Kiran C. Patel College of Osteopathic Medicine, Nova Southeastern University, Fort Lauderdale, FL 33328, USA; 2 Department of Global Health, Robert Stempel College of Public Health & Social Work, Florida International University, Miami, FL 33199, USA; alejarri@fiu.edu

**Keywords:** Medicaid expansion, patient safety culture, health reform, public health policy, hospital survey on patient safety culture (HSOPS)

## Abstract

Background: The Affordable Care Act (ACA) included provisions to improve access and patient safety. However, in some cases, improving access generated a rapid increase in demand, leading to a strain on the healthcare workforce, with potential impacts on patient safety. This study seeks to assess a potential trade-off between access and safety that could have resulted from Medicaid expansion, a key provision of ACA. To our knowledge, this is the first study that directly links Medicaid expansion to internal patient safety culture dynamics within hospitals. Methods: We used a panel data model with hospital fixed effects to assess how different dimensions of patient safety culture were affected in hospitals located in states that expanded Medicaid. We also clustered standard errors by hospital to account for dependence among responses within a single facility and correct for heteroskedasticity, thus achieving more precise and reliable estimates. Results: In small hospitals, Medicaid expansion is significantly associated with a reduced Teamwork Within Units dimension (−0.067, *p* < 0.01), a small but significant 1.7% decrease in a 1-to-5 Likert scale, while a negative association with Nonpunitive Response to Error dimension was observed in medium-sized hospitals (−0.045, *p* < 0.05) and in large hospitals (−0.064, *p* < 0.05). Conclusions: Public policies to improve healthcare access should plan around resources and capabilities to ensure patient safety. Small hospitals need additional support to manage the healthcare worker shortage that results from higher demand of healthcare services, while medium and large hospitals need to respond more in the areas of response to error.

## 1. Introduction

Healthcare systems are trending towards analyzing and understanding how to develop highly reliable organizations that aim towards zero-harm for its patients [1]. These initiatives require comprehension of the culture for patient safety within health systems. The monumental report “To Err is Human: Building a Safer Health System” declared that health systems must prioritize the culture of patient safety in order to deliver quality care in America [2]. Patient safety culture is “the product of individual and group values, attitudes, perceptions, competencies, and patterns of behavior that determine the commitment to an organization’s health and safety management” [3]. Patient safety culture affects other aspects of healthcare that can be directly or indirectly linked to patient safety [4]. It is proven to be positively associated with increased patients’ family satisfaction and patient experience, and reduced mortality, medication errors, community-acquired pneumonia, urinary tract infection, and readmission [5]. Equally as important, patient safety culture influences patient outcomes indirectly through adherence to evidence-based practices [6].

Unfortunately, healthcare workers have to endure many obstacles such as heavy workloads, burnout, high turnover, and long hours that negatively affects their work experiences [7] and overall patient safety [8,9]. A shortage in the healthcare workforce can exacerbate many of these difficulties [10].

In 2010, the United States passed the Patient Protection and Affordable Care Act (ACA). A substantial provision within this law was the expansion on Medicaid eligibility to 133% below the poverty line. While the ACA is a federal law, the additional coverage for Medicaid expansion is voluntary [11], creating the possibility to compare states that accepted the ACA’s Medicaid expansion with states that did not. In general, states that expanded Medicaid saw a rapid increase in Medicaid and private insurance coverage compared to states who did not expand Medicaid [12]. Larger coverage increased the demand for healthcare services [13,14], but the healthcare workforce did not expanded commensurately to meet this demand [15,16], adding more burden on an already depleted workforce. It was projected that forty percent of the workforce shortage in 2022 would be in relation to the ACA [17]. A similar effect was observed in Massachusetts health reform, credited as the foundation of the ACA, where nurses had a significant increase in full-time equivalent hours per patient day after the expansion of insurance coverage [18].

This study examines how Medicaid expansion was associated with changes in different dimensions of patient safety culture in hospitals. Given the increased demand for healthcare services following Medicaid expansion, we hypothesize that workforce shortages would disproportionately affect staffing and teamwork dimensions. To our knowledge, no prior studies have examined how Medicaid expansion was associated with changes in patient safety culture across U.S. hospitals. While earlier research has addressed workforce strain [7,10] and changes in patient volume following the ACA [13,14], none have linked these system-level shifts to internal hospital safety culture, a critical determinant of care quality. This study fills that gap by using a large national panel of HSOPS responses (2008–2017) to identify how Medicaid expansion may have affected specific safety culture dimensions by hospital size. It also contributes to the limited literature on how public policies influence internal hospital dynamics such as safety culture, when these dynamics interact with variations in hospital size. Smaller hospitals may be uniquely vulnerable to teamwork disruptions under rising workload pressures, whereas larger institutions may experience shifts in safety culture due to more complex oversight structures. By examining these heterogeneous effects, this study provides a better understanding of how organizational context shapes the response to major health policy reforms.

## 2. Methods

We used the Hospital Survey on Patient Safety Culture (HSOPS) Version 1.0, an instrument developed by the Agency for Healthcare Research and Quality (AHRQ) in 2004. Version 1 of HSOPS includes 42 items grouped into 12 dimensions of patient safety culture (composites). These composites include Communication Openness, Feedback and Communication About Error, Frequency of Events Reported, Handoffs and Transitions, Management Support for Patient Safety, Nonpunitive Response to Error, Organizational Learning-Continuous Improvement, Overall Perceptions of Patient Safety, Staffing, Supervisor/Manager Expectations and Actions Promoting Patient Safety, Teamwork Across Units, and Teamwork Within Units. The survey is distributed to all staff, with direct or indirect contact with patients, and administered via paper, web or mixed mode based on the hospitals’ capabilities. The participants answer survey questions that are answered in a Likert scale (Strongly Disagree = 1, Disagree = 2, Neutral = 3, Agree = 4, Strongly Agree = 5), with the addition of a single question asking the respondent to give their organization a letter grade between A and E. Respondents also filled in information describing their demographics in reference to the hospital, such as how long they have work at the facility, and their working unit [3].

All items that were negatively worded were reversed to make them comparable to positively worded items. We explored the association of Medicaid expansion on each item within these composites, by hospital size. HSOPS defines bed sizes categories as follows: 6–24 beds = 1, 25–49 beds = 2, 50–99 beds = 3, 100–199 beds = 4, 200–299 beds = 5, 300–399 beds = 6, 400–499 beds = 7, 500 or more beds = 8 [19]. However, for this study we interpreted and labeled bed size categories based on the classification of the American Hospital Association (small hospitals: under 100 beds, medium hospitals: 100–399 beds, large hospitals: 400 or more beds) [20], a widely used national-hospital statistics source.

Several categorical variables were used to capture the workers’ characteristics. Time worked in the hospital and on the unit hospital was classified into six categories (less than 1 year, 1–5 years, 6–10 years, 11–15 years, 16–20 years, and more than 20 years). Weekly hours worked were divided into six ranges: less than 20 h, 20–39 h, 40–59 h, 60–79 h, 80–99 h, and 100 h or more. Furthermore, job position was classified into different hierarchical roles within the hospital, from nurses to administrative and medical staff, and grouped and coded to assess their impact on patient safety. In addition, the length of time worked in the worker’s specialty or profession is measured, also classified in years, and a quadratic version of this variable was generated to capture potential nonlinear effects. A binary variable on direct patient interaction was also included, where “yes” indicates that the worker has contact with patients and “no” indicates that they do not. These categorical variables allow for a precise assessment of the relationship between experience and patient safety in the hospital setting.

Before describing the detailed econometric model, we provide a brief overview of our analytical approach. Our goal was to compare hospitals in states that expanded Medicaid with those in states that did not, and to observe how their reported patient safety culture changed over time. To do this, we used the HSOPS responses from 2008 to 2017 and followed hospitals across multiple survey waves. This approach allows us to isolate changes occurring after Medicaid expansion while holding constant characteristics of each hospital that do not change over time. We also accounted for differences in staff roles, experience, and working conditions to ensure that our comparisons reflect associations with Medicaid expansion rather than differences in work-force composition.

The study employs a panel data model estimated by least squares with fixed effects, to control hospital-level characteristics that do not change over time. The hospital fixed effect (H_h_) removes the influence of time-invariant factors such as hospital location, academic affiliation, and baseline patient safety culture scores. We also include a time trend (T_t_) to capture trends in patient safety culture across all hospitals. To ensure robust inference, standard errors are corrected by clustering at the hospital level, which corrects for both heteroskedasticity and correlation of observations within each health center.

The policy intervention variable was Medicaid expansion (M_ht_), a binary indicator of whether hospital *h* was in a state that expanded Medicaid in year *t* when the survey was administered. Because states adopted Medicaid expansion at different times, our model leverages this staggered rollout by using a two-way fixed-effects difference-in-differences design. The Medicaid expansion indicator (M_ht_) switches from 0 to 1 in the year a hospital’s state implemented expansion, allowing us to compare patient safety culture before and after expansion within hospitals, while non-expansion states provide contemporaneous controls. This approach follows the standard econometric framework for staggered adoption policies [21,22,23].

Because HSOPS data is anonymized at the individual level, we follow hospitals over time rather than tracking individual respondents across survey years, although individual responses are captured. Equation (1) below describes our specific econometric model, which includes hospital fixed effects (H_h_) and a time trend (T_t_). Our goal is to estimate γ, which captures the association between Medicaid expansion and a particular composite of patient safety culture Y_iht_.

(1) $$Y_{iht}=\beta_{0}+\gamma \;\cdot M_{ht}+\beta_{1}X_{iht}+\beta_{2}Z_{ht}+\beta_{3}H_{h}+\beta_{4}T_{t}+\varepsilon_{iht}$$

As stated, our goal is to estimate γ in Equation (1), which represents the association between Medicaid expansion and a given composite of patient safety culture Y_iht_ as reported by individual *i* in hospital *h* at time *t*. It should be noted that this patient safety dimension is created with the simple average of its different items (regressions will be made by dimension and by items).

To account for potential confounders, we control for individual characteristics (X_iht_, staff occupation, tenure, and occupational tenure) and time-varying hospital characteristics (Z_ht_), including bed size, number of staff, and services offered. The error term, ϵ_iht_, captures unobserved variation in responses. All data was de-identified and provided by AHRQ. The study received IRB exemption number IRB-22-0030 from Florida International University.

## 3. Results

The analysis included approximately 1,739,085 observations, corresponding to staff from 1747 hospitals between the years 2008 and 2017 (Table 1). Out of those observations, 458,933 respondents (across 513 hospitals) were in states that accepted or were in the process of accepting Medicaid expansion.

Table 2 shows the results of the association between Medicaid expansion and two dimensions of patient safety culture by hospital size. Our results show that Teamwork Within Units was negatively affected in all hospitals but was statistically significant for small hospitals. There was a negative and statistically significant association on three of four items: People support one another in this unit (−0.083, *p* < 0.01), When a lot of work needs to be done quickly, we work together as a team to get the work done (−0.067, *p* < 0.05), In this unit, people treat each other with respect (−0.076, *p* < 0.01). The total coefficient for the Teamwork Within Units represents a decrease of 0.067 on the 1 to 5 scale after the implementation of Medicaid expansion. This is equivalent to 1.7% reduction in the full scale (about one-sixth of a category step), which is small in magnitude but statistically significant.

In mid-sized hospitals, a negative and significant association was observed in one dimension, Nonpunitive Response to Error (−0.045, *p* < 0.05). In these hospitals, Medicaid expansion may have negatively affected staff perceptions of fairness in error management. Furthermore, two of the three items in this dimension were significant: When an event is reported, it feels like the person is being written up, not the problem (−0.037, *p* < 0.05), and Staff worry that mistakes they make are kept in their personnel file (−0.056, *p* < 0.01).

Table 2 also shows the results for large hospitals. There is an association in only one dimension, Nonpunitive Response to Error (−0.064, *p* < 0.05). Within the items in this dimension, there is the following association: Staff worry that mistakes they make are kept in their personnel file (−0.099, *p* < 0.01), reflecting a strong concern on the part of workers about their errors being recorded in their personnel files, which could have long-term consequences, especially after the Medicaid expansion.

In the same way, the control variables will be analyzed, these results are found in the Appendix A (Table A1, Table A2 and Table A3). It can be observed that, considering all hospital sizes, in almost all dimensions, healthcare personnel with more years of experience in their unit have a more positive response compared to those with longer experience in the hospital, who have more negative responses regarding patient safety. Furthermore, the longer the work hours (more than 60 h per week), the more negative their perceptions are in different safety dimensions. Regarding occupations, administrators/management have more positive perceptions than nurses, and physicians are the occupation with the second highest number of positive responses. Years of experience in their profession have a nonlinear association, suggesting that initially, more years of experience are associated with lower self-esteem, but that this association reverses after a certain point, when greater experience begins to have a positive association. Finally, a general positive trend is observed in all dimensions. It is worth noting that the results by items follow the same trend (see Appendix A).

## 4. Discussion

### 4.1. Statement of Principal Findings

This study suggests a potential trade-off between increased healthcare access and patient safety culture, particularly among smaller hospitals. The decline observed in teamwork-related composites likely reflects the structural vulnerability of these facilities. Smaller institutions typically operate with limited staffing reserves and reduced operational slack, which makes them more susceptible to disruptions when patient volume increases. Under these conditions, even modest increases in workload can strain informal coordination, impede mutual support, and weaken the relational dynamics that underpin strong teamwork. This interpretation aligns with prior evidence demonstrating that staffing adequacy and nurse work environments are closely linked to teamwork, engagement, and patient safety outcomes [24,25].

In contrast, medium and large hospitals did not experience a similar decline in teamwork but instead exhibited significant reductions in nonpunitive response to error following Medicaid expansion. This pattern suggests a different organizational mechanism. Larger hospitals often possess more complex oversight structures, multilayered reporting systems, and formal accountability processes. As patient volumes increase, these institutions may intensify monitoring or tighten performance expectations to manage operational pressures. While such actions may aim to maintain safety, they can inadvertently heighten staff concerns about blame, reputational risk, or punitive consequences when reporting errors. This aligns with qualitative evidence showing that fear of negative repercussions remains a major barrier to voluntary error reporting in highly structured and hierarchical clinical environments [26,27,28].

Taken together, these findings highlight that Medicaid expansion may have interacted with hospital capacity in distinct ways: intensifying workload-related teamwork challenges in smaller hospitals while amplifying concerns about punitive responses to errors in larger ones. These differentiated organizational responses underscore the need for tailored strategies to preserve safety culture across hospitals of varying size during periods of increased demand. Given these size-specific patterns, future research should examine patient safety culture through a more granular lens, exploring how structural capacity, workforce composition, and organizational processes interact differently across small, medium, and large hospitals.

### 4.2. Strengths and Limitations

This study has several notable strengths. First, it uses a comprehensive and internationally validated database, such as the HSOPS, which ensures the quality and comparability of the information on patient safety culture. Furthermore, an econometric approach with fixed effects and clustering by hospital was applied, allowing for control of time-invariant factors and improving the internal validity of the analysis. The inclusion of detailed individual-level variables, such as job title, years of experience, and patient contact, allowed for capturing heterogeneity in perceptions. Potential nonlinear effects were also explored by including quadratic terms, which enriches the analysis of professional experience. Finally, the use of an exogenous policy, such as Medicaid expansion, as a variable of interest adds relevance and pertinence to the study.

However, the study also has some limitations. The impossibility of tracking the same individuals over time due to data anonymity makes it difficult to capture personal dynamics and control for constant individual characteristics. Furthermore, although multiple factors were controlled for, some relevant variables, such as workload or the organizational climate specific to each unit, were not included. There is also a risk that Medicaid expansion may be correlated with other cofounders such as state-level policies or broader ACA impacts, which may bias the estimates. For this reason, the results should be interpreted as associations rather than causal effects.

Despite these limitations, this study provides valuable insights into how Medicaid expansion may have differentially influenced patient safety culture based on hospital size. The findings contribute to a broader understanding of how policy-driven changes can impact organizational culture and highlight the need for tailored interventions to support hospitals during periods of significant policy transition.

### 4.3. Interpretation Within the Context of the Wider Literature

This study adds to the growing literature that explores the unintended consequences of healthcare policies on hospital operations, particularly patient safety culture. While much of the existing research focuses on the financial and access-related benefits of Medicaid expansion, few studies have examined its impact on internal organizational dynamics like patient safety culture [29]. These findings extend prior evidence on the ACA’s impact on healthcare workforce strain [15,17] by showing that the resulting pressures may also influence internal cultural domains such as teamwork and nonpunitive error response. Our results partially align with studies documenting that increased workloads and burnout can impair teamwork [8,9]. However, the differentiated associations by hospital size observed here have not been previously reported. Medium and large hospitals showed stronger deterioration in nonpunitive response to error, consistent with literature suggesting that highly structured and hierarchical organizations may inhibit open reporting [26,27,28]. Conversely, declines in teamwork were more concentrated in small hospitals, extending evidence that resource constraints exacerbate operational challenges in smaller facilities.

### 4.4. Implications for Policy, Practice and Research

Medicaid expansion has significantly improved healthcare access, reducing the number of uninsured individuals and increasing patient volumes across hospitals. The findings from this study suggest that Medicaid expansion may have contributed to workforce strain in smaller hospitals while affecting perceptions of nonpunitive response to errors in larger hospitals.

For small hospitals, workforce shortages and resource constraints may have played a role in the decline in teamwork culture. These hospitals often operate with limited staffing, making it more difficult to manage higher patient volumes efficiently. Policymakers should consider targeted workforce incentives, such as educational interventions, loan forgiveness programs for rural providers and financial grants to support hiring and retention in smaller hospitals [30]. Research has shown that investment in workforce development can help reduce staff shortages and improve patient safety, particularly in hospitals with fewer resources [31]. Expanding telemedicine services may also help smaller hospitals manage increased demand more effectively without overburdening their existing workforce [32].

For medium and large hospitals, the increase in concerns about punitive responses to errors suggests that, while these facilities may be better equipped to handle increased patient loads, they may have also introduced stricter oversight measures that unintentionally discouraged open reporting. Large hospitals often have more complex administrative structures, which can make it difficult to balance accountability with a culture of safety. Research has shown that leadership engagement and commitment in safety training programs can improved communication channels [33], while transparent reporting policies can strengthen hospital safety culture and improve patient outcomes [34].

Future research should focus on the organizational factors driving these shifts in patient safety culture. Longitudinal studies can help determine whether these trends persist over time or if hospitals successfully adjust. Additionally, qualitative research on perceptions with hospital staff and leadership could provide insight into the workplace dynamics contributing to these shifts. Understanding why some hospitals maintain a strong safety culture post-expansion while others struggle can help shape best practices for future healthcare policy reforms.

## 5. Conclusions

This study offers important insights into how health-related policies, such as Medicaid expansion depicted in this study, may affect hospital operations, particularly regarding patient safety culture. While these policies have increased healthcare access, this study highlights the need for hospitals to adapt to the operational demands that accompany such changes. In smaller hospitals, teamwork showed signs of strain, and larger hospitals experienced challenges related to a nonpunitive response to errors. These findings suggest that while the overall impact of these policies on access is clear, operational adjustments and additional support for hospitals are necessary to sustain strong patient safety cultures.

These insights underscore the importance of proactive planning and resource allocation to ensure that hospitals, especially smaller facilities, can meet increased demand without compromising safety or care quality. By addressing these operational challenges, hospitals can continue to deliver safe, effective care in the evolving healthcare landscape shaped by policy reforms like Medicaid expansion. Further research is essential to develop strategies that support hospitals in managing these complexities and maintaining high standards of patient care.

## Figures and Tables

**Table 1:** Total Descriptive Statistics.

	Medicaid Expansion States	Non-Medicaid Expansion States	Total
Total Number of Hospitals	513 *	1521 *	1747
Total Number of Observations	458,933	1,280,152	1,739,085

* Includes hospitals in states that changed from non-expansion to expansion over the years (the same hospitals can be in both groups).

**Table 2:** Association of Medicaid expansion with items in patient safety dimensions.

Dimensions	Hospital Size		
	Small	Medium	Large
Teamwork Within Units	−0.067 **	−0.012	−0.002
People support one another in this unit	−0.083 **	−0.018	−0.023
When a lot of work needs to be done quickly, we work together as a team to get the work done.	−0.067 *	−0.008	−0.003
In this unit, people treat each other with respect	−0.076 **	−0.006	−0.001
When one area in this unit gets really busy, others help out	−0.038	−0.012	0.014
Nonpunitive Response to Error	−0.016	−0.045 *	−0.064 *
Staff feel like their mistakes are held against them	−0.006	−0.040	−0.043
When an event is reported, it feels like the person is being written up, not the problem.	−0.011	−0.037 *	−0.046
Staff worry that mistakes they make are kept in their personnel file.	−0.03	−0.056 **	−0.099 **

* *p* < 0.05, ** *p* < 0.01.

## Data Availability

Data may be obtained from a third party and are not publicly available. Deidentified participant data—Contact: SOPSResearchData westat. com—Website: https://www.ahrq.gov/sops/databases/research-datasets.html (accessed on 1 March 2020)—Reuse: Must send data request form to above contact. The SOPS data used in this analysis was provided by the SOPS Database. The SOPS Database is funded by the US Agency for Healthcare Research and Quality (AHRQ) and administered by Westat under Contract Number HHSP233201500026I/HHSP23337004T.

## Appendix A

This appendix presents full estimation results for each of the twelve patient safety culture dimensions for small (Table A1), medium (Table A2) and large hospitals (Table A3). For three patient safety culture dimensions, the Medicaid expansion indicator was statistically significant: Teamwork Within Units, Management Support (for small hospitals), and Nonpunitive Response to Errors (for medium and large hospitals). Table A4, Table A5 and Table A6 present full estimation results for each item that compose those 3 patient safety culture dimensions, for small, medium and large hospitals, respectively.

**Table A1:** Association of Medicaid Expansion with Patient Safety Culture Dimensions for Small Hospitals.

Variables	Teamwork Within Units	Supervisor Leadership	Organizational Learning	Management Support	Overall Perceptions	Comm. About Error	Communication Openness	Reporting Frequency	Teamwork Across Units	Staffing	Handoffs & Transitions	Nonpunitive Response
Medicaid Expansion	−0.067 **	−0.034	−0.028	−0.047	−0.018	−0.025	−0.036	−0.029	−0.022	−0.013	0.007	−0.016
Years in Unit	0.023 **	0.026 **	0.029 **	0.017 **	0.029 **	0.028 **	0.032 **	0.019 **	0.014 **	0.030 **	−0.010 **	0.026 **
Years in hospital	−0.033 **	−0.046 **	−0.020 **	−0.028 **	−0.039 **	−0.036 **	−0.039 **	−0.025 **	−0.022 **	−0.046 **	−0.008 **	−0.044 **
Less than 20 h per week (omitted)												
20 to 59 h per week	−0.107 **	−0.093 **	−0.041 **	−0.103 **	−0.099 **	−0.094 **	−0.119 **	−0.003	−0.147 **	−0.104 **	−0.162 **	−0.143 **
60 or more hours per week	−0.176 **	−0.166 **	−0.042 *	−0.160 **	−0.187 **	−0.109 **	−0.169 **	0.006	−0.178 **	−0.241 **	−0.230 **	−0.255 **
Nurse (omitted)												
Patient Care Asst	−0.144 **	0.020 *	0.036 **	0.164 **	0.079 **	0.159 **	−0.033 **	0.157 **	0.074 **	−0.127 **	0.054 **	−0.129 **
Physician	0.179 **	0.085 **	0.071 **	0.211 **	0.195 **	0.066 **	0.174 **	−0.113 **	0.200 **	0.027	0.041 *	0.038
Pharmacist/Dietitian	0.069 **	0.198 **	0.165 **	0.219 **	0.268 **	0.259 **	0.257 **	−0.071 **	0.193 **	0.208 **	−0.062 **	0.313 **
Unit Assistant	−0.064 **	0.102 **	0.032 **	0.252 **	0.229 **	0.202 **	0.037 **	0.154 **	0.114 **	−0.003	0.123 **	−0.066 **
Therapist/Technician	−0.017	0.078 **	0.029 **	0.208 **	0.305 **	0.142 **	0.086 **	0.028 **	0.110 **	0.112 **	0.040 **	0.043 **
Admin/Management	0.291 **	0.392 **	0.329 **	0.543 **	0.476 **	0.405 **	0.388 **	0.149 **	0.330 **	0.313 **	0.221 **	0.506 **
Other	−0.054 **	0.071 **	0.015	0.278 **	0.226 **	0.164 **	0.034 **	0.075 **	0.134 **	−0.053 **	0.089 **	−0.032 **
Direct interaction	−0.016 *	−0.033 **	−0.021 **	−0.074 **	−0.005	−0.040 **	−0.036 **	−0.048 **	−0.022 **	0.036 **	0.056 **	−0.038 **
Years in Profession	−0.116 **	−0.096 **	−0.096 **	−0.144 **	−0.102 **	−0.134 **	−0.114 **	−0.021 *	−0.143 **	−0.005	−0.136 **	−0.070 **
Years in Profession^2^	0.017 **	0.015 **	0.013 **	0.021 **	0.015 **	0.018 **	0.017 **	0.005 **	0.020 **	0.004 **	0.018 **	0.011 **
Year	0.016 **	0.023 **	0.008 **	0.008 *	0.007 *	0.020 **	0.023 **	0.018 **	0.017 **	−0.003	0.014 **	0.033 **
Constant	−26.946 **	−42.058 **	−11.251 *	−11.463	−9.483	−36.32 **	−41.73 **	−33.18 **	−31.11 **	10.139	−23.95 **	−62.66 **

* *p* < 0.05, ** *p* < 0.01.

**Table A2:** Association of Medicaid Expansion with Patient Safety Culture Dimensions for Medium Hospitals.

Variables	Teamwork Within Units	Supervisor Leadership	Organizational Learning	Management Support	Overall Perceptions	Comm. About Error	Comm. Openness	Reporting Frequency	Teamwork Across Units	Staffing	Handoffs & Transitions	Nonpunitive Response
Medicaid Expansion	−0.012	0.016	−0.004	0.005	−0.005	−0.003	−0.014	−0.003	−0.002	−0.017	−0.006	−0.045 *
Years in Unit	0.017 **	0.023 **	0.024 **	0.013 **	0.022 **	0.025 **	0.029 **	0.017 **	0.013 **	0.027 **	−0.009 **	0.016 **
Years in hospital	−0.027 **	−0.045 **	−0.018 **	−0.030 **	−0.041 **	−0.037 **	−0.040 **	−0.027 **	−0.019 **	−0.045 **	−0.004 **	−0.041 **
Less than 20 h per week (omitted)												
20 to 59 h per week	−0.116 **	−0.098 **	−0.052 **	−0.117 **	−0.127 **	−0.089 **	−0.109 **	−0.007	−0.136 **	−0.102 **	−0.133 **	−0.121 **
60 or more hours per week	−0.180 **	−0.146 **	−0.051 **	−0.157 **	−0.194 **	−0.087 **	−0.144 **	0.012	−0.158 **	−0.223 **	−0.196 **	−0.199 **
Nurse (omitted)												
Patient Care Asst	−0.170 **	0.041 **	0.059 **	0.230 **	0.104 **	0.205 **	−0.008	0.204 **	0.116 **	−0.115 **	0.117 **	−0.112 **
Physician	0.136 **	0.094 **	0.037 **	0.137 **	0.154 **	−0.02	0.144 **	−0.193 **	0.121 **	0.036 **	−0.067 **	0.128 **
Pharmacist/Dietitian	−0.080 **	0.033 **	0.039 **	0.096 **	0.084 **	0.059 **	0.061 **	−0.206 **	0.058 **	0.082 **	−0.189 **	0.168 **
Unit Assistant	−0.076 **	0.109 **	0.027 **	0.278 **	0.235 **	0.206 **	0.052 **	0.187 **	0.150 **	0.031 **	0.170 **	−0.044 **
Therapist/Technician	−0.073 **	0.020 **	−0.026 **	0.179 **	0.245 **	0.059 **	0.008	0.012 *	0.090 **	0.077 **	0.034 **	−0.039 **
Admin/Management	0.257 **	0.388 **	0.283 **	0.514 **	0.448 **	0.368 **	0.364 **	0.156 **	0.287 **	0.265 **	0.155 **	0.451 **
Other	−0.082 **	0.053 **	−0.017 **	0.267 **	0.211 **	0.105 **	0.001	0.053 **	0.143 **	−0.041 **	0.104 **	−0.030 **
Direct interaction	0.014 **	−0.021 **	−0.003	−0.069 **	0.001	−0.003	−0.005	−0.033 **	0.003	0.038 **	0.061 **	−0.016 **
Years in Profession	−0.166 **	−0.134 **	−0.124 **	−0.186 **	−0.152 **	−0.159 **	−0.147 **	−0.050 **	−0.163 **	−0.041 **	−0.139 **	−0.100 **
Years in Profession^2^	0.022 **	0.019 **	0.016 **	0.027 **	0.021 **	0.020 **	0.021 **	0.008 **	0.023 **	0.009 **	0.019 **	0.015 **
Year	0.015 **	0.018 **	0.005 *	0.001	0.002	0.018 **	0.014 **	0.015 **	0.013 **	−0.015 **	0.011 **	0.024 **
Constant	−26.815 **	−31.548 **	−6.253	2.354	0.234	−32.73 **	−24.80 **	−26.66 **	−21.863 **	32.69 **	−17.68 **	−45.139 **

* *p* < 0.05, ** *p* < 0.01.

**Table A3:** Association of Medicaid Expansion with Patient Safety Culture Dimensions for Large Hospitals.

Variables	Teamwork Within Units	Supervisor Leadership	Organizational Learning	Management Support	Overall Perceptions	Comm. About Error	Comm. Openness	Reporting Frequency	Teamwork Across Units	Staffing	Handoffs & Transitions	Nonpunitive Response
Medicaid Expansion	−0.002	−0.04	−0.013	0.016	−0.002	−0.022	−0.028	0.006	0.019	0.025	0.008	−0.064 *
Years in Unit	0.009 **	0.022 **	0.021 **	0.009 **	0.018 **	0.023 **	0.029 **	0.015 **	0.011 **	0.029 **	−0.010 **	0.016 **
Years in hospital	−0.025 **	−0.046 **	−0.021 **	−0.033 **	−0.043 **	−0.040 **	−0.044 **	−0.027 **	−0.021 **	−0.048 **	−0.006 *	−0.041 **
Less than 20 h per week (omitted)												
20 to 59 h per week	−0.125 **	−0.090 **	−0.048 **	−0.117 **	−0.108 **	−0.074 **	−0.109 **	0.004	−0.116 **	−0.093 **	−0.110 **	−0.113 **
60 or more hours per week	−0.195 **	−0.142 **	−0.050 **	−0.158 **	−0.177 **	−0.097 **	−0.156 **	0.02	−0.134 **	−0.216 **	−0.181 **	−0.189 **
Nurse (omitted)												
Patient Care Asst	−0.172 **	0.069 **	0.071 **	0.263 **	0.132 **	0.208 **	−0.003	0.218 **	0.133 **	−0.088 **	0.135 **	−0.121 **
Physician	0.136 **	0.116 **	0.046 **	0.123 **	0.178 **	−0.059 **	0.165 **	−0.255 **	0.107 **	0.112 **	−0.074 **	0.185 **
Pharmacist/Dietitian	−0.115 **	−0.003	0.016	0.049 *	0.025	−0.047 *	−0.014	−0.243 **	0	0.005	−0.241 **	0.091 **
Unit Assistant	−0.094 **	0.110 **	0.026	0.304 **	0.221 **	0.189 **	0.047 **	0.188 **	0.172 **	0.042 **	0.183 **	−0.081 **
Therapist/Technician	−0.101 **	−0.001	−0.040 **	0.170 **	0.206 **	0.014	−0.032 **	0.009	0.086 **	0.050 **	0.048 **	−0.094 **
Admin/Management	0.223 **	0.357 **	0.253 **	0.494 **	0.403 **	0.303 **	0.311 **	0.135 **	0.256 **	0.191 **	0.137 **	0.383 **
Other	−0.085 **	0.045 **	−0.012	0.272 **	0.200 **	0.065 **	−0.014	0.050 **	0.145 **	−0.030 *	0.136 **	−0.059 **
Direct interaction	0.031 **	−0.014	0.02	−0.066 **	−0.006	0.013	0.008	−0.031 *	0.007	0.032 *	0.044 **	−0.027 *
Years in Profession	−0.187 **	−0.155 **	−0.136 **	−0.210 **	−0.178 **	−0.177 **	−0.167 **	−0.069 **	−0.163 **	−0.066 **	−0.131 **	−0.120 **
Years in Profession^2^	0.024 **	0.022 **	0.018 **	0.030 **	0.025 **	0.023 **	0.023 **	0.011 **	0.022 **	0.012 **	0.018 **	0.017 **
Year	0.018 *	0.043 **	0.008	−0.002	0.004	0.026 **	0.031 **	0.005	0.01	−0.027 *	0.014	0.040 **
Constant	−31.276 *	−81.620 **	−11.189	8.947	−3.445	−47.909 *	−59.39 **	−6.636	−16.267	56.807 **	−25.2	−77.11 **

* *p* < 0.05, ** *p* < 0.01.

**Table A4:** Association of Medicaid expansion with patient safety culture items in small hospitals.

	Teamwork Within Units				Management Support for Patient Safety			Nonpunitive Response to Error		
Variables	TW1	TW2	TW3	TW4	SP1	SP2	SP3	NR1	NR2	NR3
Medicaid Expansion	−0.083 **	−0.067 *	−0.076 **	−0.038	−0.077 **	−0.044	−0.025	−0.006	−0.011	−0.03
Years in Unit	0.024 **	0.026 **	0.030 **	0.011 **	0.017 **	0.017 **	0.016 **	0.028 **	0.038 **	0.013 **
Years in hospital	−0.031 **	−0.026 **	−0.047 **	−0.029 **	−0.030 **	−0.025 **	−0.029 **	−0.047 **	−0.046 **	−0.039 **
Less than 20 h per week (omitted)										
20 to 59 h per week	−0.108 **	−0.062 **	−0.165 **	−0.095 **	−0.103 **	−0.098 **	−0.111 **	−0.160 **	−0.103 **	−0.170 **
60 or more hours per week	−0.184 **	−0.126 **	−0.250 **	−0.146 **	−0.149 **	−0.114 **	−0.218 **	−0.273 **	−0.228 **	−0.273 **
Nurse (omitted)										
Patient Care Asst	−0.171 **	−0.179 **	−0.138 **	−0.093 **	0.180 **	0.208 **	0.097 **	−0.122 **	−0.140 **	−0.120 **
Physician	0.171 **	0.084 **	0.312 **	0.148 **	0.232 **	0.218 **	0.180 **	0.047	0.022	0.041
Pharmacist/Dietitian	0.025	0.02	0.061 **	0.163 **	0.205 **	0.242 **	0.207 **	0.264 **	0.297 **	0.364 **
Unit Assistant	−0.107 **	−0.072 **	−0.094 **	0.015	0.265 **	0.271 **	0.219 **	−0.068 **	−0.063 **	−0.057 **
Therapist/Technician	−0.051 **	−0.037 **	−0.027 *	0.047 **	0.236 **	0.218 **	0.165 **	0.034 **	0.025 *	0.069 **
Administration/Management	0.259 **	0.262 **	0.291 **	0.346 **	0.540 **	0.553 **	0.528 **	0.490 **	0.579 **	0.445 **
Other	−0.113 **	−0.070 **	−0.072 **	0.041 **	0.308 **	0.308 **	0.213 **	−0.044 **	−0.034 **	−0.009
Direct interaction	−0.013	−0.008	−0.034 **	−0.014	−0.063 **	−0.081 **	−0.077 **	−0.051 **	−0.044 **	−0.029 **
Years in Profession	−0.117 **	−0.071 **	−0.114 **	−0.162 **	−0.128 **	−0.154 **	−0.147 **	−0.071 **	−0.049 **	−0.088 **
Years in Profession^2^	0.017 **	0.011 **	0.017 **	0.022 **	0.018 **	0.022 **	0.024 **	0.012 **	0.010 **	0.012 **
Year	0.015 **	0.010 **	0.021 **	0.016 **	0.012 **	0.009 *	0.002	0.029 **	0.029 **	0.041 **
Constant	−25.994 **	−16.704 **	−37.855 **	−28.102 **	−20.688 **	−13.096	−0.333	−54.151 **	−55.773 **	−78.483 **

* *p* < 0.05, ** *p* < 0.01.

**Table A5:** Association of Medicaid expansion with patient safety culture items in Medium hospitals.

	Teamwork Within Units				Management Support for Patient Safety			Nonpunitive Response to Error		
Variables	TW1	TW2	TW3	TW4	SP1	SP2	SP3	NR1	NR2	NR3
Medicaid Expansion	−0.018	−0.008	−0.006	−0.012	−0.001	0.017	0	−0.040	−0.037 *	−0.056 **
Years in Unit	0.017 **	0.020 **	0.025 **	0.006 **	0.013 **	0.013 **	0.011 **	0.021 **	0.025 **	0.001
Years in hospital	−0.024 **	−0.019 **	−0.042 **	−0.025 **	−0.035 **	−0.025 **	−0.028 **	−0.045 **	−0.040 **	−0.037 **
Less than 20 h per week (omitted)										
20 to 59 h per week	−0.114 **	−0.073 **	−0.180 **	−0.095 **	−0.129 **	−0.109 **	−0.114 **	−0.138 **	−0.086 **	−0.140 **
60 or more hours per week	−0.190 **	−0.131 **	−0.260 **	−0.143 **	−0.158 **	−0.120 **	−0.194 **	−0.218 **	−0.166 **	−0.215 **
Nurse (omitted)										
Patient Care Asst	−0.206 **	−0.219 **	−0.157 **	−0.100 **	0.239 **	0.284 **	0.162 **	−0.118 **	−0.128 **	−0.093 **
Physician	0.141 **	0.051 **	0.279 **	0.073 **	0.162 **	0.117 **	0.132 **	0.137 **	0.083 **	0.165 **
Pharmacist/Dietitian	−0.110 **	−0.128 **	−0.056 **	−0.029 *	0.105 **	0.123 **	0.059 **	0.154 **	0.167 **	0.175 **
Unit Assistant	−0.131 **	−0.072 **	−0.103 **	0.002	0.297 **	0.293 **	0.244 **	−0.051 **	−0.048 **	−0.030 **
Therapist/Technician	−0.104 **	−0.066 **	−0.059 **	−0.065 **	0.218 **	0.185 **	0.132 **	−0.052 **	−0.044 **	−0.020 *
Administration/Management	0.231 **	0.247 **	0.272 **	0.269 **	0.525 **	0.516 **	0.494 **	0.429 **	0.533 **	0.380 **
Other	−0.138 **	−0.089 **	−0.066 **	−0.027 **	0.309 **	0.288 **	0.203 **	−0.049 **	−0.029 **	−0.004
Direct interaction	0.025 **	0.003	0.005	0.017 **	−0.052 **	−0.071 **	−0.083 **	−0.025 **	−0.022 **	−0.014 *
Years in Profession	−0.175 **	−0.118 **	−0.169 **	−0.201 **	−0.175 **	−0.191 **	−0.187 **	−0.105 **	−0.086 **	−0.111 **
Years in Profession^2^	0.023 **	0.016 **	0.023 **	0.025 **	0.024 **	0.027 **	0.030 **	0.016 **	0.014 **	0.015 **
Year	0.015 **	0.011 **	0.019 **	0.017 **	0.004	0.003	−0.004	0.019 **	0.022 **	0.032 **
Constant	−25.258 **	−16.851 **	−33.708 **	−30.440 **	−3.924	−2.058	12.703	−33.883 **	−40.581 **	−60.970 **

* *p* < 0.05, ** *p* < 0.01.

**Table A6:** Association of Medicaid expansion with patient safety culture items in large hospitals.

	Teamwork Within Units				Management Support for Patient Safety			Nonpunitive Response to Error		
Variables	TW1	TW2	TW3	TW4	SP1	SP2	SP3	NR1	NR2	NR3
Medicaid Expansion	−0.023	−0.003	0	0.014	0.004	0.015	0.024	−0.043	−0.046	−0.099 **
Years in Unit	0.008 **	0.013 **	0.013 **	0.003	0.009 **	0.012 **	0.006	0.020 **	0.024 **	0.004
Years in hospital	−0.020 **	−0.017 **	−0.037 **	−0.026 **	−0.040 **	−0.029 **	−0.031 **	−0.045 **	−0.037 **	−0.040 **
Less than 20 h per week (omitted)										
20 to 59 h per week	−0.126 **	−0.087 **	−0.197 **	−0.090 **	−0.120 **	−0.120 **	−0.105 **	−0.135 **	−0.083 **	−0.124 **
60 or more hours per week	−0.206 **	−0.154 **	−0.281 **	−0.140 **	−0.145 **	−0.136 **	−0.188 **	−0.226 **	−0.162 **	−0.186 **
Nurse (omitted)										
Patient Care Asst	−0.211 **	−0.209 **	−0.166 **	−0.109 **	0.285 **	0.320 **	0.181 **	−0.117 **	−0.141 **	−0.107 **
Physician	0.139 **	0.060 **	0.292 **	0.049 **	0.146 **	0.084 **	0.142 **	0.195 **	0.129 **	0.231 **
Pharmacist/Dietitian	−0.134 **	−0.150 **	−0.080 **	−0.108 **	0.066 **	0.078 **	0.002	0.088 **	0.111 **	0.073 *
Unit Assistant	−0.154 **	−0.104 **	−0.112 **	−0.015	0.324 **	0.317 **	0.268 **	−0.085 **	−0.083 **	−0.070 **
Therapist/Technician	−0.129 **	−0.089 **	−0.090 **	−0.094 **	0.210 **	0.175 **	0.123 **	−0.106 **	−0.093 **	−0.085 **
Administration/Management	0.207 **	0.226 **	0.251 **	0.207 **	0.520 **	0.490 **	0.468 **	0.366 **	0.467 **	0.315 **
Other	−0.142 **	−0.092 **	−0.059 **	−0.047 **	0.313 **	0.287 **	0.214 **	−0.071 **	−0.061 **	−0.041 *
Direct interaction	0.042 **	0.017	0.012	0.047 **	−0.050 **	−0.065 **	−0.082 **	−0.037 **	−0.033 *	−0.017
Years in Profession	−0.204 **	−0.137 **	−0.197 **	−0.207 **	−0.205 **	−0.215 **	−0.203 **	−0.130 **	−0.104 **	−0.123 **
Years in Profession^2^	0.027 **	0.019 **	0.026 **	0.025 **	0.028 **	0.029 **	0.032 **	0.019 **	0.016 **	0.016 **
Year	0.022 **	0.013	0.027 **	0.01	0.003	−0.003	−0.006	0.026	0.030 **	0.061 **
Constant	−39.953 **	−22.586	−50.388 **	−15.551	−2.261	9.375	15.266	−49.284	−57.210 **	−118.845 **

* *p* < 0.05, ** *p* < 0.01.
